# Genetic diversity within and genetic differentiation between blooms of a microalgal species

**DOI:** 10.1111/j.1462-2920.2012.02769.x

**Published:** 2012-09

**Authors:** Karen Lebret, Emma S Kritzberg, Rosa Figueroa, Karin Rengefors

**Affiliations:** Aquatic Ecology, Department of Biology, Lund UniversityEcology Building, Lund SE-223 62, Sweden

## Abstract

The field of genetic diversity in protists, particularly phytoplankton, is under expansion. However, little is known regarding variation in genetic diversity within populations over time. The aim of our study was to investigate intrapopulation genetic diversity and genetic differentiation in the freshwater bloom-forming microalga *Gonyostomum semen* (*Raphidophyceae*). The study covered a 2-year period including all phases of the bloom. Amplified fragment length polymorphism (AFLP) was used to determine the genetic structure and diversity of the population. Our results showed a significant differentiation between samples collected during the two blooms from consecutive years. Also, an increase of gene diversity and a loss of differentiation among sampling dates were observed over time within a single bloom. The latter observations may reflect the continuous germination of cysts from the sediment. The life cycle characteristics of *G. semen*, particularly reproduction and recruitment, most likely explain a high proportion of the observed variation. This study highlights the importance of the life cycle for the intraspecific genetic diversity of microbial species, which alternates between sexual and asexual reproduction.

## Introduction

The field of genetic diversity in microorganisms has been under expansion since the development of recent molecular tools. Yet, little is known about the mechanisms involved in the generation and maintenance of genetic diversity in microbes in general, and about the dynamics of populations in particular. Due to their short generation time and huge population size, microbes are interesting and suitable organisms when studying the mechanisms involved in genetic differentiation and population dynamics. In addition, microorganisms show an extreme diversity, and live in a large variety of environments. In aquatic systems, autotrophic protists can make up an important part of the biomass and are major contributors to primary production. Some species can form blooms, which can be considered as large ephemeral populations because they occur within a few weeks or months (Dolan, 2005).

Bloom-forming phytoplanktonic species have been intensely studied in their ecology, the environmental conditions enhancing the bloom formation, and capacity to overgrow other species. Nevertheless, few studies have focused on investigating their intraspecific genetic diversity. Indeed, due to their largely asexual proliferation and low morphological variation, phytoplankton blooms have often been assumed to be either monoclonal or of extremely low genetic diversity. However, several recent studies have shown surprisingly high intraspecific genetic diversity in both marine and limnic species (Hayhome *et al*., 1987; John *et al*., 2004; Evans *et al*., 2005; Rynearson and Armbrust, 2005; Logares *et al*., 2009). Population genetic studies of phytoplankton over time reveal the existence of sexual events, and also the potential differentiation of populations caused by differences in abiotic conditions. Genetic differences in populations can lead to phenotypic variations that are important for the physiological and ecological characteristics of a given species. In addition, a species with high genetic diversity may be able to respond and adapt faster to environmental changes.

Lake phytoplankton is of special interest in population genetics as they are microbial in size [and thereby presumably disperse easily (Finlay, 2002)] yet consist of populations that are isolated (by land mass) from each other. Lakes are considered to act like islands (as in the theory of island biogeography; MacArthur and Wilson, 1967) due to their spatial isolation (Dodson, 1992). Hence, a limited gene flow is expected between lake populations, especially for lakes located far apart. Consequently, sexual and life cycle events may have a greater impact on the genetic diversity within lake populations than in marine systems where populations are expected to be more homogeneous given that they are more connected to each other, by water currents for instance.

To date, studies concerning phytoplankton and genetic diversity have mostly focused on spatial scales. These studies showed genetic differentiation over space in several marine and freshwater species at small and largescales (Coleman, 2001; Rynearson and Armbrust, 2004; Nagai *et al*., 2007; Godhe and Härnström, 2010). Although autotrophic protists have relatively short generation times, which could result in rapid differentiation of populations over time, only a handful studies have highlighted temporal changes in genetic diversity and structure (Evans *et al*., 2005; Rynearson *et al*., 2006; 2009; Godhe and Härnström, 2010). Temporal differentiation in phytoplankton can be the results of variation in environmental conditions (Rynearson *et al*., 2006; 2009) or sexual events (Evans *et al*., 2005).

In this study we have focused on *Gonyostomum semen* (Ehrenberg) Diesing – a nuisance freshwater raphidophyte, responsible for dense blooms that can dominate the phytoplankton community by up to 95% (Cronberg *et al*., 1988; Pithart *et al*., 1997; Willén, 2003; Findlay *et al*., 2005). This species was previously known as a rare species restricted to a few small humic lakes around the world. Over the last four decades, its occurrence has increased, and it now forms blooms in a wider range of lakes in Scandinavia, Finland and eastern Baltic countries, including larger non-humic lakes (Cronberg *et al*., 1988; Lepistö*et al*., 1994). This raphidophyte is not known to be toxic, in contrast to its marine relatives. However, it produces slimy mucilage, which may cause allergic reactions on the skin of bathers, making some Scandinavian lakes unattractive for recreational purposes (Cronberg *et al*., 1988; Lepistö*et al*., 1994; Findlay *et al*., 2005).

The *G. semen* life cycle has been well described through both field and laboratory observations and experiments (Cronberg, 2005; Figueroa and Rengefors, 2006). *Gonyostomum semen* proliferates asexually through cell division in the water column throughout the summer season. At the end of the bloom, the cells typically undergo sexual reproduction, which is immediately followed by resting cyst formation. *Gonyostomum semen* overwinters as cysts in the sediment. In the spring, the cysts germinate and form flagellated vegetative cells that migrate back into the water column.

The aim of our study was to investigate the genetic diversity and population structure of *G. semen* over time during two consecutive blooms, to include all the different bloom stages (encystment, germination, exponential growth, sexual events and encystment). We used amplified fragment length polymorphism (AFLP) (Vos *et al*., 1995), which has previously been used to distinguish different strains of *G. semen* (Figueroa and Rengefors, 2006). The technique has been successfully applied in previous population genetic studies of non-model organisms (e.g. Bensch and Åkesson, 2005), including phytoplankton species (De Bruin *et al*., 2003). AFLP has shown high reproducibility (Jones *et al*., 1997), and is therefore highly suitable for large population studies. First, we hypothesized that intraspecific genetic diversity varies over time following changes of the environmental conditions and life cycle characteristics of the species. We expected a decrease in diversity over time with a single or few genotypes becoming dominant. The alternative hypothesis was an increase in genetic diversity due to either recruitment of new genotypes from the sediments or sexual reproduction. Our second hypothesis was that only a single population would be observed throughout the season since no barriers to gene flow are expected within one lake. Finally, the third hypothesis was that the two consecutive blooms would show no genetic differentiation, as no change in allele frequency was expected following sexual reproduction.

## Results

### *G. semen* abundance and bloom

**Fig. 1:**
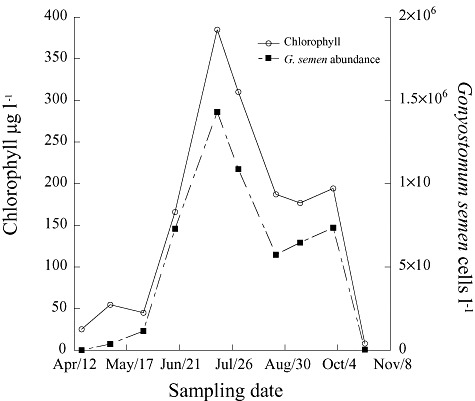
Chlorophyll *a* concentration (open circles) and *G. semen* abundance (black square) over time during the 2009 bloom.

In 2009, vegetative cells of *G. semen* appeared in the water column in April (Fig. 1). *Gonyostomum semen* formed a dense bloom with a maximum in July reaching almost 1.5 million cells l^−1^ and a chlorophyll *a* maximum of 385 µg l^−1^. *Gonyostomum semen* was the most abundant phytoplankton species in Lake Bökesjö, and comprised more than 80% of the phytoplankton biomass between June and October with a maximum of 97% in July. The bloom ended in October with a rapid decrease of *G. semen* cell number in the water column. During the sampling season, the lake was stratified with an anoxic layer below 2 m depth until the last sampling date (1 October 2009) when the water column was mixed.

### Isolation success

A total of 182 strains were successfully isolated, cultured without cyanobacterial or protist contamination (although not axenically) and genotyped (Table 1). The survival rates ranged from 21% to 75%.

**Table 1:** Number of strains isolated for each sampling date, the percentage of survival and number of strains genotyped for the study

Year of sampling	Sampling date	Number of cells isolated	Percentage of survival	Number of strains genotyped
2008	1 October	12	75	4
	10 October	24	58	6
	14 October	48	40	10
	24 October	48	44	7
2009	17 April	90	57	19
	5 May	69	42	10
	28 May	69	38	20
	18 June	89	37	13
	16 July	72	49	19
	30 July	72	54	19
	24 August	96	52	20
	9 September	96	43	20
	1 October	95	21	15

### Genetic diversity within sampling dates

**Fig. 2:**
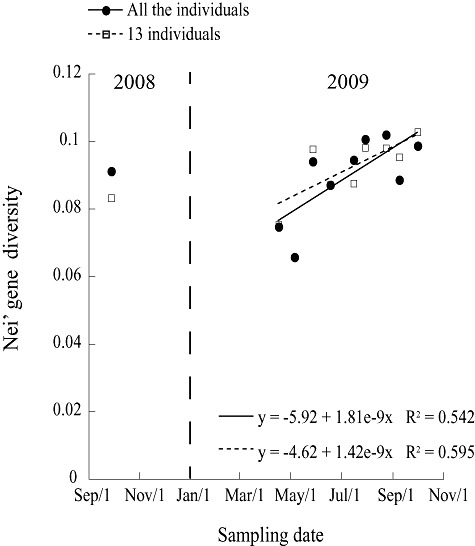
Nei's gene diversity over time, in 2008 and during 2009 bloom of *G. semen*, with all the individuals (circles with full line) and the data set including 13 individuals per sampling date (squares with the dotted line).

After scoring the raw data, 337 loci were retained for the analysis. The AFLP profiles of all the individual clonal isolates were non-identical, i.e. no clones were re-sampled during the season. The percentage of polymorphic loci ranged from 18% to 41% per sampling date (Table 2). The Nei's gene diversity of *G. semen* clonal isolates for each sampling date ranged between 0.066 and 0.102. In 2009, the gene diversity increased during the bloom (Fig. 2), from 0.075 in April to 0.099 in October (*R*^2^ = 0.542, *P* = 0.024). The October value of gene diversity was similar to those observed at the end of the bloom in 2008 (Fig. 2). To determine the effect of unequal sample sizes of the different sampling dates, gene diversity was calculated on a reduced data set including 13 individuals for each sampling date. For an equal number of individuals per sampling date, the percentage of polymorphic loci ranged from 24% to 35% (Table 2), and Nei's gene diversity ranged between 0.075 and 0.103. The correlation between Nei's gene diversity over time (in 2009 only) was still observed (*R*^2^ = 0.595; *P* = 0.012; Fig. 2).

**Table 2:** Percentage of polymorphic loci and Nei's gene diversity with the 95% confidence interval (C.I.) for each sampling date

Year of sampling	Sampling date	All individuals			13 individuals per sampling date		
	
		Percentage of variable markers	Nei's gene diversity	95% C.I.	Percentage of variable markers	Nei's gene diversity	95% C.I.
2008	October	41%	0.091	0.069–0.098	28%	0.081	0.064–0.098
2009	17 April	29%	0.075	0.053–0.081	26%	0.075	0.060–0.092
	6 May	18%	0.066	0.045–0.074	–	–	–
	28 May	35%	0.094	0.069–0.100	32%	0.098	0.081–0.116
	18 June	24%	0.087	0.062–0.096	24%	0.087	0.070–0.106
	16 July	35%	0.094	0.069–0.100	27%	0.088	0.069–0.107
	30 July	36%	0.101	0.073–0.107	31%	0.098	0.081–0.117
	24 August	36%	0.102	0.075–0.109	30%	0.098	0.080–0.116
	9 September	33%	0.089	0.064–0.096	30%	0.095	0.078–0.114
	1 October	36%	0.099	0.073–0.104	35%	0.103	0.085–0.122

### Genetic differentiation between sampling dates

*F*_ST_ values, estimates of the genetic differentiation between sampling dates, ranged between 0.003 and 0.180 (Table 3). Highly significant differences (*P* < 0.001) were observed between samples collected in 2008 and samples from all the sampling dates of 2009 with values ranging from 0.103 to 0.180 (Table 3). Individuals from the first sampling date in 2009, April 17th, were significantly different (*P* < 0.01) from all other sampling dates, and *F*_ST_ values ranged from 0.061 to 0.130. The only exception was the second date of sampling, which was not significantly different from the first sampling date. The last five dates of sampling during the bloom did not show significant genetic differentiation between each other.

**Table 3:** Pair-wise *F*_ST_ between each sampling dates and level of significance

	October 2008	17 April 2009	6 May 2009	28 May 2009	18 June 2009	16 July 2009	30 July 2009	24 August 2009	9 September 2009	1 October 2009
October 2008	0.000									
17 April 2009	0.160***	0.000								
6 May 2009	0.140***	−0.004^ns^	0.000							
28 May 2009	0.115***	0.117***	0.065*	0.000						
18 June 2009	0.180***	0.130***	0.107*	0.019^ns^	0.000					
16 July 2009	0.160***	0.065**	0.067*	0.047*	0.013^ns^	0.000				
30 July 2009	0.154***	0.073**	0.055^ns^	0.048*	0.007^ns^	0.004^ns^	0.000			
24 August 2009	0.103***	0.067**	0.026^ns^	0.007^ns^	0.014^ns^	0.018^ns^	−0.006^ns^	0.000		
9 September 2009	0.098***	0.061**	0.016^ns^	0.034*	0.082***	0.059*	0.042*	0.003^ns^	0.000	
1 October 2009	0.105***	0.058*	0.052*	0.013^ns^	0.013^ns^	0.001^ns^	−0.004^ns^	−0.007^ns^	0.026^ns^	0.000

ns, not significant; * significant with *P* < 0.05, ** significant with *P* < 0.01, *** significant with *P* < 0.001.

### Population structure

**Fig. 3:**
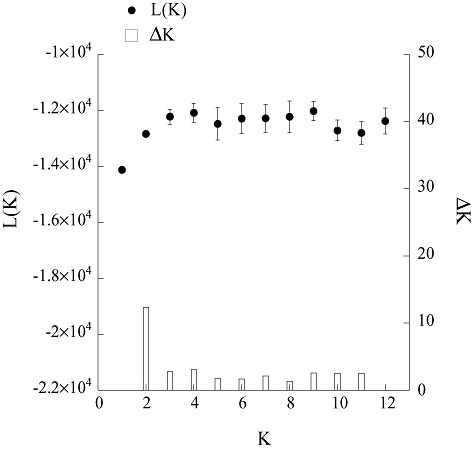
Summary of the results from the STRUCTURE analysis using the no-admixture ancestry model allowing for independent frequencies, L(K) (black dots) and ΔK (bars) for the different K population assumptions.

The population structure analyses performed using the software STRUCTURE suggested the presence of a single population. Using the Evanno and colleagues' (2005) method, a very small peak of ΔK was detected at K equal 2 (ΔK = 12.34, for the no-admixture and independent frequency model: Fig. 3), indicating the possibility of two populations. However, on the likelihood curve (L(K)), the plateau was already reached at K equal 2, suggesting that there was only one population. Given the weak support for two populations with the Evanno method, we concluded that a model with one population best explained the data.

## Discussion

In this study, for the first time, we describe changes in genetic diversity during frequent intervals over time in a single lake phytoplankton bloom, as well as between two consecutive blooms. Despite a predominance of asexual reproduction, we observed an increase in genetic diversity coupled with a loss of genetic differentiation between samples over time. In addition, we found significant genetic differentiation between the populations sampled for two consecutive years in the same lake.

### Genetic diversity and clonal polymorphism

The genotypic diversity observed for each sampling date concurs with previous studies demonstrating that phytoplankton blooms are not monoclonal events (Hayhome *et al*., 1987; Rynearson and Armbrust, 2000; 2004; 2005; John *et al*., 2004; Logares *et al*., 2009; Alpermann *et al*., 2010; Lowe *et al*., 2010). In addition, we showed that genetic diversity did not decline as a bloom develops, which further discards the notion that blooms are dominated by a few clones. As previously shown in both marine and limnic environments, no dominance of a highly adapted and fast-growing clone over less competitive ones was observed (Lowe *et al*., 2010). Rynearson and Armbrust (2005) estimated that at least 2400 genetically distinct lineages composed a bloom of the diatom *Ditylum brightwellii*, showing the extreme diversity occurring within a single bloom. In the same study the *D. brightwellii* population had similar genetic diversity values as observed for the *G. semen* population in the current study. If we assume a similar genotypic diversity for *G. semen* as the one observed for *D. brightwellii*, the probability of isolating two identical clones with our sampling design (20 cells per sampling date) was very low, which is in accordance with our results.

The level of genetic diversity in a population is a result of the population's history. In our study, the history of our specific lake population is unknown; however, the recent species history suggests that *G. semen* might be under expansion (Cronberg *et al*., 1988; Lepistö*et al*., 1994; Rengefors *et al*., 2012). The genetic diversity values in this *G. semen* population might be low compared with other organisms; however, considering that the species might be under expansion, relatively low levels of genetic diversity should be expected. Many rare mutations would be expected, i.e. alleles with low frequencies, which is exactly what we observed in this system. Also, studies concerning genetic diversity in phytoplankton and protists in general are still scarce, thus the levels and range of genetic diversity of these organisms are still unclear and require further investigation.

In addition, a study by Figueroa and Rengefors (2006) describing the life cycle of *G. semen* indicated that *G. semen* might be homothallic. Homothallism implies that the organism can potentially reproduce sexually by self-fertilization. Thus inbreeding may happen in homothallic species, leading to populations with low genetic diversity. The relatively low gene diversity observed in the *G. semen* population may thus be partly explained by the homothallic characteristics of the *G. semen* life cycle.

The true genetic diversity in phytoplankton populations may be underestimated, as there is a bias during the isolation step. Isolation involves a selection of the genotypes that are able to grow in the culture conditions imposed on them (Gallagher, 1980; Lakeman *et al*., 2009). To minimize this potential selection, we used a medium prepared with 50% of lake water, which increased the survival rates of the isolates in the lab. The survival rates were on average greater than or equal to 40%, which is a reasonable proportion as *G. semen* cells are extremely sensitive to any mechanical disturbances. The latest date of sampling showed a lower survival, which may be caused by the fact that the cells were isolated from a declining bloom in which most cells were forming cysts.

### Genetic differentiation 2008/2009

Highly significant differentiation (*F*_ST_) was observed between the isolates from 2008 and 2009, of similar magnitude as the differentiation measured between populations of *D. brightwellii* sampled from different sites 20 km apart by Rynearson and colleagues (2006) using microsatellite markers. For comparison, the differentiation observed between the two consecutive years was of the same magnitude as that between freshwater fish in different lakes observed in recent studies (Sønstebø*et al*., 2007; Wang *et al*., 2007). Thus, temporal differentiation of *G. semen* within Lake Bökesjö occurred at a range similar to the differentiation occurring between populations in different lakes or sampling locations in phytoplankton species and also in some macroorganisms.

Temporal differentiation of phytoplanktonic populations has been observed in previous studies, mostly in marine diatoms. In contrast to the present study, these studies have focused on marine species and had long time intervals between sampling. For instance, Evans and colleagues (2005) and Godhe and Härnström (2010) showed that small genetic differentiations were observed over time for the marine diatoms *Pseudo-nitzschia pungens* and *Skeletonema marinoi* respectively. Rynearson and colleagues (2006; 2009) distinguished two subpopulations of the diatom *D. brightwellii* collected the same year, occurring at the same sampling site but collected few months apart. This differentiation was explained by environmental selection, as the external conditions were different when the two subpopulations occurred. Logares and colleagues (2009) found that strains isolated from the same population of dinoflagellates (*Peridiniopsis borgei*) clustered in separate populations mainly corresponding to the year they were isolated.

Like many phytoplankton species, *G. semen* produces resting cysts after sexual reproduction, which overwinter in the sediment in order to survive winter conditions that are unfavourable for vegetative growth (Cronberg, 2005; Figueroa and Rengefors, 2006). The cysts formed in 2008 presumably contributed massively to the initiation of the 2009 bloom. The resting stages in the sediment are considered as storage of genetic diversity, as they accumulate every year, and stay viable for several years. As soon as the conditions are favourable for germination this genetic diversity is liberated to the water column (Fryxell, 1983). Alpermann and colleagues (2009) showed that a bloom of the dinoflagellate *Alexandrium tamarense* was composed of cells that had emerged from the germination of cysts of different year classes, thus generating a high genetic diversity within a single bloom. In our study, cysts formed prior to 2008 may also have contributed to the differentiation but this effect is likely minor, as the majority of these cysts would have germinated already or died. The mortality of *G. semen* cysts is likely high comparable to that observed with freshwater dinoflagellate cysts [50% or more during the first year (Rengefors, 1998)]. Moreover, most germination occurs from the top layer of the sediment, which in turn likely contains a majority, but not exclusively, of newly formed cysts (Rengefors, 1998). Nevertheless, we cannot rule out that part of the differentiation observed between the 2008 and 2009 populations may be explained by recruitment from an older cyst bank.

Sexual reproduction cannot explain the differentiation observed between the 2 years, as sexual events *per se* would not have caused changes in the allele frequencies. We have two suggestions of potential explanations for the observed differentiation. The first is that a high mortality of overwintering cysts may have resulted in a change in allele frequencies. This shift could be caused either by random mortality or by a strong selective mortality. The other explanation is that environmental conditions might have differed between the years, leading to a selection of different genotypes that germinate and grow. However, further studies are necessary to clarify this issue.

### Genetic diversity variation over time

During the *G. semen* bloom, an increase in genetic diversity was observed over time. This pattern was observed independently of the number of sampled individuals, as the correlations were significant and had similar trends using all the individuals or the data set including the same number of individuals per sampling date. Thus, in our study, the variation in sample size did not significantly influence our results. The large number of loci genotyped most probably limited the bias that can be produced by unequal sample sizes. As previously mentioned, the measured genetic diversity might underestimate the real diversity in the lake, because the measured diversity was calculated using the strains able to grow with the conditions imposed on them through culturing.

The increase of genetic diversity in the cultured strains was mostly caused by low values at the beginning compared with the end of 2009 bloom. There are two possible explanations for the low genetic diversity at the beginning of the bloom. One is high mortality of cysts during over-wintering periods as observed in dinoflagellates (Rengefors, 1998), which may have led to an important loss of genetic diversity. Mortality of cysts would have needed to be associated with mutations in the surviving genotypes to permit the observed increase of genetic diversity during the bloom, and by its end reached values similar to 2008. However, this increase could not be caused by mutations only, as the number of generations between April and October was only 14, if we consider one generation every 13 days (calculated from *G. semen* growth rate between 28 May and 16 July 2009). Nor can it be explained by genetic recombination, as there was no evidence of sexual reproduction (i.e. presence of fusing cells) until September. Instead, an alternative explanation is that the increase of genetic diversity is caused by a continuous germination of new genotypes throughout the spring and early summer. In a field study, Hansson (2000) observed that the recruitment of *G. semen* from the sediment (resting stages) to the water column can occur during the entire growth season. In that study, *G. semen* cells were observed in recruitment traps until September. On the other hand, germination experiments (Rengefors *et al*., 2012) indicate that cysts germinate rapidly at temperatures above 10°C, suggesting that the cyst bank should be quickly exhausted once summer water temperatures have been reached.

The constant germination of cysts, contributing new genotypes, may be the key factor allowing both the increase of the genetic diversity and the loss of genetic differentiation among the sampling dates over time. The increase in genetic diversity was the highest between the second and third sampling dates, suggesting that the germination of cysts mostly occurred in April–May, which corresponds with the conclusions that can be drawn from the laboratory study on germination (Rengefors *et al*., 2012). Germination may still occur after this period but had a more negligible effect on the genetic diversity. New germination events may occur, for instance, when non-germinated cysts are transported to the surface because of bioturbation, or when a new area of the sediment becomes oxidized. Other explanations include differences in dormancy period or differential responses to changes in temperature by different genotypes. The germination of resting stages in dinoflagellates (which have a similar life cycle to raphidophytes) is regulated by the dormancy period, in occasions an endogenous clock, and temperature (Rengefors and Anderson, 1998). Similarly, *G. semen* is also regulated by a dormancy period (2 months), an endogenous clock and temperature (Rengefors *et al*., 2012).

### Genetic differentiation within the bloom

In addition to the differentiation observed between the two consecutive blooms, a highly significant differentiation within the 2009 bloom was found between the samples collected during the first sampling date and the later sampling dates. This indicates a change in the genetic structure of the population at the beginning of the bloom. Mutation alone cannot explain differentiation occurring within a few weeks, since this mechanism needs a longer timescale to be detected at the population level. As the change occurred within a population over a short time, the differentiation could be the result of either a change towards genotypes better adapted to the local conditions, or the addition of several new genotypes in the water column following either recruitment or colonization events. Either of these events could have induced changes in the genetic structure of the population. A change towards dominance by one or few genotypes would in theory have been associated with a decrease in gene diversity, which was not observed in the current study. Thus, the hypothesis that the differentiation was caused by a change towards a dominance of a few well-adapted genotypes was rejected. On the other hand, recruitment or colonization events would have led to an increase of the gene diversity due to the emergence of new genotypes in the population. The appearance of new genotypes in the populations through dispersal and colonization is probably negligible in the observed differentiation, and hence we attribute the differentiation to recruitment of new genotypes from cysts.

### *G. semen* population structure within lake

In our study, the Bayesian population analysis by STRUCTURE identified one population. Thus the STRUCTURE analysis did not discriminate different populations despite high *F*_ST_ values (some *F*_ST_ > 0.15) with high significance. Halkett and colleagues (2005) suggested that STRUCURE analysis might not be ideal to study populations reproducing mainly asexually, as the Hardy-Weinberg equilibrium assumption might not be observed for some microbial populations. Also, whereas *F*_ST_ analyses heterozygosity between pre-defined populations, STRUCTURE does not use prior information concerning the samples. Hence, STRUCTURE analysis is often considered more conservative, and detects a higher level of population structure.

### Conclusions

This study addresses fine-scale temporal differences both within and between consecutive blooms. With the strains successfully isolated and cultivated, we found significant genetic differentiation between two consecutive populations of a microalga within a single lake. Furthermore, we observed variation of genetic diversity during the bloom along with differentiation between sampling dates, possibly due to the germination of resting cysts, leading to an increase of genetic diversity over time. We propose that for phytoplankton, intrapopulation genetic diversity may be closely related to their life cycle. Studies on different phytoplankton groups are necessary to understand how life cycle can have large implications on genetic diversity patterns over time. Also, caution should be taken when interpreting data from comparative studies on diverse populations as the bloom state of the populations must be considered before drawing any conclusions.

## Experimental procedures

### Study site

*Gonyostomum semen* cells were collected in Lake Bökesjö (Skåne, Sweden) at 55°34′N, 13°26′E. Lake Bökesjö is a small humic lake (surface area 0.015 km^2^, at an altitude of 65 m, 4.5 m maximum depth) situated in a beech forest in the nature reserve Häckeberga (Gaillard *et al*., 1996). *Gonyostomum semen* is known to occur regularly in this lake and forms dense blooms every year (K. Rengefors, unpubl. data).

### Sampling

To compare the population genetic structure of two consecutive years, sampling was performed in October 2008 (at the end of the *G. semen* bloom), and between April and October 2009. In 2008, samples were taken from the shore on four occasions within 3 weeks, for cell isolation only. In 2009, to cover all the phases of the bloom, samples were collected on nine occasions with 3-week intervals. Sampling started in April, corresponding to the initiation of cyst germination, and ended in October when the resting cysts were formed and the bloom ended. At the deepest point of the lake, a HQ30d temperature/oxygen meter (Hach) was used to measure the temperature and oxygen concentration of the water column in order to determine the depth of the stratification. Samples for chlorophyll *a* analysis and phytoplankton abundance were collected in the epilimnion using a plexiglass tube sampler (2 m long, volume 1.8 l). For *G. semen* isolation, in October 2008, the cells were collected from the shore in the surface water using a 20 µm net. In 2009, during low abundance of *G. semen* (< 1000 *G. semen* cells ml^−1^), a fresh sample of phytoplankton was collected using a 20 µm net in the epilimnion. For the sampling during high abundance of *G. semen* (> 1000 *G. semen* cells ml^−1^), a water sample of the epilimnion column was collected using the plexiglass tube sampler, as *G. semen* clogged the net when present in high abundance.

### Chlorophyll *a* analysis and *G. semen* abundance

The epilimnetic water was filtered through GF/C glass fibre filter for chlorophyll *a* determination. Samples were processed according Jespersen and Christoffersen (1987). The *G. semen* abundance was determined by cell counts at a 40× magnification (the cells are large, ∼ 50 µm) using an inverted microscope (Nikon Eclipse TS100). Epilimnetic water samples were collected and preserved with Lugol's solution. For the early and late phase of the bloom (low abundance of *G. semen*), 20 ml of sample was sedimented in a 20 ml counting chamber overnight. At high abundance, *G. semen* cells were counted in 1 ml of sample using a Sedgewick-Rafter counting chamber, the whole chamber was counted.

### Isolation, culture and harvesting of *G. semen* cells

Single cell isolations were performed to obtain a final number of approximately 20 clonal cultures per sampling date (Table 1). The isolations of *G. semen* vegetative cells were done in the laboratory using an inverted microscope (Nikon Eclipse TS100) and a glass micropipette prepared with 100 µl capillary tubes (Hirschmann Laborgeräte, Germany). The cells were transferred and washed three times in a mix of50% of sterile filtered Bökesjö water and 50% of artificial MWC medium (Guillard and Lorenzen, 1972) modified by an addition of selenium (1.84 mg l^−1^). Subsequently, the cells were transferred to individual wells of a 96-well Nunc plate (Nunclon, Denmark) containing 200 µl of medium–lake water mix.

The strains were cultivated in increasing volumes of mix 50% MWC-50% sterile filtered Bökesjö-water at 20°C with a light 20 µmol m^−2^ s^−1^, with 12 h:12 h light : dark cycle. When the cell concentration had reached approximately 2000 cells ml^−1^, 20 ml of culture was harvested by centrifugation at 500 *g* for 15 min. The pellets were transferred to a 1.5 ml tube and centrifuged at 500 *g* for 10 min and the medium was discharged. The pellets were frozen at −80°C until DNA extraction.

### DNA extraction

DNA extraction was performed using a CTAB-base method with the following steps. The pellets of cells were resuspended with 0.7 ml of CTAB lysis buffer [100 mM Tris-HCl (pH 8), 1.4 M NaCl, 20 mM EDTA, 2% (w/v) cetyltrimethylammonium bromide (CTAB), 0.4% (v/v) β-mercaptoethanol, 1% (w/v) polyvinylpyrollidone (PVP) (Dempster *et al*., 1999)]. The samples were incubated at 65°C for 60 min, and mixed regularly. To extract the DNA, 0.7 ml of chloroform : isoamyl alcohol (24:1) was added to the samples. The samples were shaken vigorously for 20 min on an orbital shaker, and then centrifuged at 20 000 *g* for 15 min at 4°C. The upper layer was transferred to a 1.5 ml tube, one volume of 5 M NaCl and two volumes of ice-cold isopropanol were added. The DNA was precipitated by centrifugation at 20 000 *g* for 15 min at 4°C. The pellet was washed twice with ice-cold ethanol (70%) and centrifugation at 20 000 *g* for 5 min at 4°C. The ethanol was discharged and the samples were dried. The DNA was resuspended with 50 µl of TE buffer. The DNA concentration of the samples was estimated by measuring absorbance of a subsample diluted 10 times at 260 nm using a (Ultraspec 3000, Pharmacia biotech). For each sample, the quality of the DNA was determined using the 260/280 ratio. Only samples of high DNA quality, i.e. with a 260/280 ratio of 2.0, were used for downstream analyses. The DNA samples were stored at −80°C until genotyping.

### Genotyping by AFLP analysis

Amplified fragment length polymorphism (AFLP) analyses were performed on the samples according to Vos and colleagues (1995) and Logares and colleagues (2009) with 125 ng of extracted DNA. For the selective amplification, the M and E-primer were 5′-GACTGCGTACCAATTCNNN-3′ and 5′-GATGAGTCCTGAGTAANNN-3′ respectively. Specifically the following six primer combinations were used: E_TCT_ × M_CGA_, E_TCT_ × M_CCG_, E_TAG_ × M_CGG_, E_TCG_ × M_CAG_, E_TCG_ × M_CGG_ and E_TCG_ × M_CGA_. The E-primers of the combinations were labelled with Ned, Fam or Hex fluorescent dyes. PCR products from three primer combinations labelled with the different dyes were combined in one single well of a 96-well plate (Applied Biosystems). The samples were diluted 10 times, and dried at room temperature before sending them for capillary electrophoresis analysis. All the samples were then analysed with a MapMarker 1000 bp size standard with an ABI37730XL capillary electrophoresis machine at the Uppsala Genome Center, Sweden. To check for the effect of the presence of bacteria on the AFLP genotyping, a few samples containing only the supernatant (containing bacteria) of *G. semen* culture were extracted and genotyped. A few AFLP peaks were observed in the samples containing only the supernatant. These fragments were not observed in the genotype of the *G. semen* strain from which the supernatant was removed. These results indicate that the bacteria content did not affect the genotyping of *G. semen* strains.

### AFLP data analyses

The raw data were analysed with Genemapper (Version 4.0, Applied Biosystems) and AFLPscore version 1.4 (Whitlock *et al*., 2008) was used to score the data. Fragments between 50 and 1000 bp. were sized and scored. The error rate between replicates was minimized to less than 5% for each primer combination based on duplicates of 20 randomly chosen strains according to Whitlock and colleagues (2008). After the scoring, a data set based on presence/absence of fragments was generated using AFLPscore. The strains isolated in October 2008 were considered as one unique sampling date due to the small interval of time between each sampling dates, to facilitate the comparison with the 2009 isolates and to obtain reasonable sampling size for the analysis. All the 2008 samples were collected within a time frame of less than 3 weeks. Also, no genetic differentiation was detected between the four sampling dates of October 2008. The data were checked manually to identify identical clones (genotypic diversity). Nei's gene diversity (Nei, 1987) and the percentage of polymorphic loci were determined for each sampling date using the R script AFLPdat (Ehrich, 2006) with all the genotyped individuals. To identify potential bias caused by differences of number of individuals per sampling date, Nei's gene diversity and percentage of polymorphic loci were determine on a reduced data set including 13 randomly chosen individuals per sampling date. The change of Nei's gene diversity during the 2009 bloom was determined by calculating the Pearson correlation between the gene diversity values over time using SPSS 19. FAMD version 1.25 (Schluter and Harris, 2006) was used to convert the data file into an input file compatible for Arlequin. Arlequin version 3.5.1.2 (Excoffier *et al*., 2005) was used to calculate pair-wise *F*_ST_ values to estimate genetic differentiation between sampling dates, the *P*-values were determined using 1000 permutations, and the *F*_ST_ were considered significant for *P* < 0.05. The number of genetic populations were determined using the software STRUCTURE 2.3.3 (Pritchard *et al*., 2000) without prior information on the date of sampling. All the combinations of model settings, admixture or no-admixture ancestry models with either correlated or independent allele frequency, were tested (four models in total). Each run had a burn-in of 20 000 iterations followed by 50 000 iterations of data collections. We tested up to 12 populations (K) with 10 iterations at each level. The results were analysed according to Evanno and colleagues (2005) to identify the number of populations that best fits our data set.
